# Effect of Maternal Prepregnancy/Early‐Pregnancy Body Mass Index and Pregnancy Smoking and Alcohol on Congenital Heart Diseases: A Parental Negative Control Study

**DOI:** 10.1161/JAHA.120.020051

**Published:** 2021-05-27

**Authors:** Kurt Taylor, Ahmed Elhakeem, Johanna Lucia Thorbjørnsrud Nader, Tiffany C. Yang, Elena Isaevska, Lorenzo Richiardi, Tanja Vrijkotte, Angela Pinot de Moira, Deirdre M. Murray, Daragh Finn, Dan Mason, John Wright, Sam Oddie, Nel Roeleveld, Jennifer R. Harris, Anne‐Marie Nybo Andersen, Massimo Caputo, Deborah A. Lawlor

**Affiliations:** ^1^ Population Health Science Bristol Medical School Bristol United Kingdom; ^2^ Medical Research Council Integrative Epidemiology Unit at the University of Bristol United Kingdom; ^3^ Division of Health Data and Digitalisation Department of Genetics and Bioinformatics Norwegian Institute of Public Health Oslo Norway; ^4^ Bradford Institute for Health Research Bradford Teaching Hospitals National Health Service Foundation Trust Bradford United Kingdom; ^5^ Cancer Epidemiology Unit Department of Medical Sciences University of Turin and CPO Piemonte Turin Italy; ^6^ Department of Public and Occupational Health Amsterdam Public Health Research Institute Amsterdam University Medical Center University of Amsterdam the Netherlands; ^7^ Section for Epidemiology Department of Public Health University of Copenhagen Denmark; ^8^ The Irish Centre for Fetal and Neonatal Translational Research University College Cork Cork Ireland; ^9^ Department of Paediatrics and Child Health University College Cork Cork Ireland; ^10^ Centre for Reviews and Dissemination University of York Heslington York United Kingdom; ^11^ Department for Health Evidence Radboud Institute for Health Sciences Radboud University Medical Center Nijmegen the Netherlands; ^12^ Division of Health Data and Digitalisation Norwegian Institute of Public Health Oslo Norway; ^13^ Centre for Fertility and Health Norwegian Institute of Public Health Oslo Norway; ^14^ Translational Science Bristol Medical School Bristol United Kingdom; ^15^ Bristol National Institute for Health Research Biomedical Research Center Bristol United Kingdom

**Keywords:** congenital heart disease, negative control, risk factors, Epidemiology, Pediatrics, Pregnancy, Lifestyle, Obesity

## Abstract

**Background:**

Congenital heart diseases (CHDs) are the most common congenital anomaly. The causes of CHDs are largely unknown. Higher prenatal body mass index (BMI), smoking, and alcohol consumption are associated with increased risk of CHDs. Whether these are causal is unclear.

**Methods and Results:**

Seven European birth cohorts, including 232 390 offspring (2469 CHD cases [1.1%]), were included. We applied negative exposure paternal control analyses to explore the intrauterine effects of maternal BMI, smoking, and alcohol consumption during pregnancy, on offspring CHDs and CHD severity. We used logistic regression, adjusting for confounders and the other parent's exposure and combined estimates using a fixed‐effects meta‐analysis. In adjusted analyses, maternal overweight (odds ratio [OR], 1.15 [95% CI, 1.01–1.31]) and obesity (OR, 1.12 [95% CI, 0.93–1.36]), compared with normal weight, were associated with higher odds of CHD, but there was no clear evidence of a linear increase in odds across the whole BMI distribution. Associations of paternal overweight, obesity, and mean BMI were similar to the maternal associations. Maternal pregnancy smoking was associated with higher odds of CHD (OR, 1.11 [95% CI, 0.97–1.25]) but paternal smoking was not (OR, 0.96 [95% CI, 0.85–1.07]). The positive association with maternal smoking appeared to be driven by nonsevere CHD cases (OR, 1.22 [95% CI, 1.04–1.44]). Associations with maternal moderate/heavy pregnancy alcohol consumption were imprecisely estimated (OR, 1.16 [95% CI, 0.52–2.58]) and similar to those for paternal consumption.

**Conclusions:**

We found evidence of an intrauterine effect for maternal smoking on offspring CHDs, but no evidence for higher maternal BMI or alcohol consumption. Our findings provide further support for the importance of smoking cessation during pregnancy.

**Nonstandard Abbreviations and Acronyms**
ABCD: The Amsterdam Born Children and Their Development Study
ALSPAC: Avon Longitudinal Study of Parents and Children
BASELINE: Cork Scope Baseline Study
BiB: Born in Bradford
CA: congenital anomaly
DNBC: Danish National Birth Cohort
MoBa: Norwegian Mother, Father and Child Cohort Study
NINFEA: Nascita e Infanzia: gli Effetti dell'Ambiente (Birth and Childhood: Effects of the Environment)

**Clinical Perspective**

**What Is New?**

- Previous studies showing associations of higher maternal body mass index, smoking, and alcohol consumption in pregnancy were not able to establish causality.
- Using parental negative control analyses, our study provides stronger evidence that maternal pregnancy smoking may increase offspring congenital heart disease risk via intrauterine mechanisms, whereas it does not suggest maternal overweight or obesity increases risk.

**What Are the Clinical Implications?**

- Emphasizing the potential adverse effect of smoking on congenital heart diseases might help in supporting women of reproductive age not to start smoking and women who are smoking at the start of pregnancy to be encouraged to quit.
- Understanding the mechanisms through which maternal smoking influences congenital heart disease risk could identify novel targets for prevention beyond smoking cessation.

Congenital heart diseases (CHDs) are the most common congenital anomaly (CA), affecting 6 to 8 per 1000 live births and 10% of stillbirths, and are the leading cause of death from CAs.^1^ Many patients with CHD present with sequela from surgical intervention and late complications related to the anomaly, resulting in health problems that persist into adulthood.^2^, ^3^ The causes of CHDs are largely unknown, but intrauterine mechanisms may play a role in their underlying pathophysiological characteristics.^4^ Identifying modifiable risk factors for CHDs is important for improving causative understanding and developing preventive interventions.

Several modifiable maternal characteristics have been found to be associated with increased risk of CHDs, including maternal prepregnancy/early‐pregnancy body mass index (BMI),^5^, ^6^, ^7^ smoking,^8^ and alcohol^9^ consumption in pregnancy. Whether these are causal is unclear. A recent systematic review and meta‐analysis of the association of BMI with CHDs found that risk of CHDs was higher in those whose mothers were overweight or obese at the start of pregnancy, compared with those who were normal weight. Results for underweight mothers were not reported,^5^ but a large cohort study consisting of >2 000 000 singletons found no clear association for maternal underweight status and CHDs.^6^ These results from conventional multivariable approaches may be explained by residual confounding because of incomplete identification or adjustment for confounders. Maternal active smoking^8^ and maternal exposure to alcohol^9^ were both associated with offspring CHDs in recent meta‐analyses. However, 68% and 69% of the studies within the meta‐analyses (for maternal smoking and alcohol, respectively) did not adjust for confounders. Therefore, those studies showing associations for smoking and alcohol cannot determine whether these reflect the magnitude of a causal effect or are biased by confounding.

Negative control studies are widely used in laboratory science and in recent years have become increasingly used to explore causal effects in epidemiology.^10^ The idea behind negative control studies is that either the exposure or the outcome in the real experiment is substituted for a negative control exposure (or outcome) that is not a plausible risk factor but would have similar sources of bias or confounding as in the main experiment. In epidemiology, this approach has been primarily used for determining the extent to which hypothesized intrauterine and early life exposures might be associated with outcomes as a result of residual confounding.^10^, ^11^ Negative parental exposure control studies are used for this purpose. This involves comparing the confounder‐adjusted associations of maternal pregnancy exposures with the offspring outcome of interest to similarly adjusted associations of the same characteristics (negative controls) in the father. The assumptions of this approach are that: (1) measured and unmeasured confounders influence the exposures in the same direction and with a similar magnitude in mothers and fathers and (2) there is no plausible reason why the exposure in the father would affect the offspring outcome (or at a minimum the paternal association would be much weaker than in the mother). In the present study, we are assuming that paternal BMI, smoking, and alcohol cannot causally influence offspring CHDs through intrauterine mechanisms. Under these assumptions, if there is a causal intrauterine effect of any of the maternal pregnancy exposures, we would expect to see a maternal‐specific association, with no (or a much weaker) association with the equivalent paternal exposure. Similar associations in mothers and fathers would suggest that these are largely driven by residual confounding. It is plausible that passive smoking from fathers could influence offspring outcomes via intrauterine exposure; however, we would expect a much weaker association for fathers. As proof of concept, maternal smoking relates strongly to lower birth weight (a known causal intrauterine effect), whereas paternal smoking has a much weaker association; and when the 2 are mutually adjusted, the maternal association remains strong, whereas the weak paternal association attenuates to the null.^10^, ^12^

We aimed to explore the causal intrauterine effects of maternal pregnancy BMI, smoking, and alcohol on CHDs using data from the Horizon 2020 LifeCycle project.^13^ As well as the negative parental control study providing scope to explore residual confounding, the use of a large existing collaboration of birth cohorts adds benefit to this study in comparison to previous studies. First, both offspring with and without CHDs are from the same underlying populations and have been selected for inclusion and assessed in identical ways. Second, most studies of risk factors for CHDs are case‐control studies, and these dominate meta‐analysis results. These have advantages in that they have large numbers of CHD cases and hence greater statistical power than most cohorts, but they are prone to selection bias as response rates in controls are commonly low, and in some studies controls are selected from hospitals or clinics and do not reflect exposure status in the population from which the cases came.^14^ Furthermore, case‐control studies are susceptible to information bias because of differential recall and reporting of the exposure between cases and controls.^14^ Third, we have harmonized data on all exposures, confounders, and outcomes. Fourth, we have large numbers, with 232 390 participants in total and 2469 CHD cases. Last, the ethos of the LifeCycle collaboration is that all studies contribute to each research question unless they do not have data on either exposure or outcome, meaning publication bias is minimized.

## Methods

Requests to access the data used in this study may be sent individually to the included cohorts. We have included details on how researchers can access each cohort at the end of the article under “data access.” Materials supporting the findings of this study are available from the corresponding author on reasonable request.

### Inclusion Criteria and Participating Cohorts

This study was part of the Horizon2020 LifeCycle Project. LifeCycle is a collaboration of largely European birth cohorts that aims to determine the impact of early‐life stressors on risk of developing adverse cardiovascular/metabolic, respiratory, cognitive, and mental health outcomes (http://lifecycle‐project.eu).^13^ A LifeCycle cohort was eligible for inclusion if it had information on CHD in the offspring ascertained by any method and data on at least one of the following: (1) mother's prepregnancy/early‐pregnancy BMI, (2) maternal smoking during pregnancy, (3) maternal alcohol consumption during pregnancy, or (4) the same exposures (1–3) measured in the father at a similar time to their pregnant partners. Eligible LifeCycle cohorts could be from any geographical area and with participants from any ethnic background. In total, 7 cohorts were eligible, and all participated: ABCD (The Amsterdam Born Children and Their Development Study),^15^ ALSPAC (Avon Longitudinal Study of Parents and Children),^16^, ^17^ BASELINE (Cork Scope Baseline Study),^18^ BiB (Born in Bradford) study,^19^ DNBC (Danish National Birth Cohort) study,^20^ MoBa (Norwegian Mother, Father and Child Cohort Study),^21^, ^22^ and NINFEA (Nascita e Infanzia: gli Effetti dell'Ambiente [Birth and Childhood: Effects of the Environment]) study.^23^, ^24^ Individual cohort descriptions can be found in Data S1. We excluded multiple births from the study population because they differ from single births for CA outcomes.^25^, ^26^ Some previous studies have excluded infants with any known chromosomal or genetic defects on the assumption that modifiable risk factors are unlikely to contribute in the presence of known causes. We have not made these exclusions in our main analyses because it is plausible that CHD in children with these complex syndromes is also influenced by the modifiable exposures we explore herein. Furthermore, from a public health and clinical perspective, we believe it is useful to know effects for all CHD cases. In additional analyses, we explore whether their removal alters our main results.

### BMI, Smoking, and Alcohol Measurements

We used harmonized LifeCycle data for exposure and confounder data, with the exclusion of paternal alcohol consumption, which had not been harmonized by LifeCycle when we started this project.^27^ ABCD and BASELINE were not part of the core LifeCycle cohorts and therefore not part of phase 1 harmonized data that we used herein. We harmonized the data for these cohorts to resemble the harmonized LifeCycle variables. Cohort‐specific information on methods of data collection can be found in Table S1.

LifeCycle‐harmonized maternal BMI used measured or self‐reported prepregnancy/early‐pregnancy weight and height. Prepregnancy weight was prioritized, and if not available, the earliest pregnancy measures were used. Paternal BMI was similarly reported (by the father or their pregnant partner) or measured, and we prioritized the timing to be prepregnancy or as early as possible in their partners pregnancy. BMI was used as a continuous variable for the main analyses. In cohorts that had >100 CHD cases, we also categorized BMI as underweight (BMI <18.5 kg/m^2^), normal weight (BMI 18.5–<25 kg/m^2^), overweight (BMI 25–<30 kg/m^2^), and obese (BMI ≥30 kg/m^2^). ALSPAC, BiB study, DNBC study, and MoBa contributed to these analyses.

We used 2 LifeCycle smoking variables for maternal and paternal smoking at the time of pregnancy: (1) smoking in the first trimester (yes/no) where this was available, otherwise any smoking during pregnancy (yes/no); and (2) categorized into nonsmokers, light smokers (<10 cigarettes smoked per day), and heavy smokers (≥10 cigarettes per day) throughout the entire pregnancy. Paternal smoking was categorized as “any smoking (yes/no)” at the time of their partners pregnancy.

We used 2 LifeCycle variables for maternal alcohol consumption: (1) binary (yes/no), which like smoking prioritized the first trimester if available but was otherwise any alcohol intake during pregnancy; and (2) categorized into nondrinkers (none), light drinkers (>0 and <3 units per week), and moderate/heavy drinkers (≥3 units per week) during pregnancy. Two studies (ALSPAC and MoBa) had data on paternal alcohol consumption in pregnancy and thus were able to harmonize variables relating to paternal alcohol for this project. We generated one variable, categorized as: nondrinkers, light drinkers (>0 and <7 units per week), or moderate/heavy drinkers (≥7 units per week) (Data S2).

The rationale for prioritizing maternal pregnancy smoking and alcohol during the first trimester is because fetal cardiac development starts early in pregnancy and much of the development occurs in the first trimester.^28^ A total of 47% and 96% of mothers had measures specifically in the first trimester for smoking and alcohol, respectively.

### CHD Outcomes

Information on CHDs was retrieved from a variety of sources, depending on the cohort. ALSPAC, BiB study, DNBC study, and NINFEA study had *International Classification of Diseases, Tenth Revision* (*ICD‐10*), coded data. BASELINE had individual CHD diagnoses assigned by a cardiologist based on echocardiography. For ABCD and MoBa, we had a nonspecific CHD diagnosis (yes/no). Data in ABCD, BASELINE, DNBC study, and NINFEA study were restricted to liveborn infants, whereas ALSPAC, BIB study, and MoBa included stillbirths.

In the ABCD cohort, data on CHDs in liveborn children were obtained from 3 different sources: (1) the infant questionnaire, which was filled out by the mother at an average infant age of 12.9 weeks; (2) the questionnaire filled out by the mother at an average child age of 5.1 years; and (3) clinical data of the Youth Health Care Registration. In the ALSPAC cohort, cases were obtained from a range of data sources, including health record linkage and questionnaire data up until age 25 years following European Surveillance of Congenital Anomalies guidelines.^29^ In BASELINE, at 2 months, mothers were asked of any medical problems and/or referrals. If a baby had been referred to a specialist, he/she was checked by a cardiologist to see if he/she had results from an echocardiogram with exact diagnoses reported. Further diagnoses up until age 12 years were identified through records from the echocardiogram. In the BiB study cohort, there were 2 separate sources to identify CAs. Both sources were used in this study: (1) CAs up to 5 years of age, identified in primary care records by Bishop et al,^30^ following European Surveillance of Congenital Anomalies guidelines. *ICD‐10* codes were mapped to clinical term‐V3 codes before extraction from primary care records. (2) Data extracted from the Yorkshire and Humber CA register database. Data were *ICD‐10* coded. All of these were confirmed postnatally. In the DNBC study, all diagnoses of CAs (according to European Surveillance of Congenital Anomalies guide 1.4, sections 3.2 and 3.3) up until the age of 15 years were extracted from the Danish National Patient Register, which is linked to the cohort data.^31^, ^32^ Diagnoses were *ICD‐10* coded. These data were restricted to children born alive. In MoBa, information on whether a child had a CHD or not was obtained though linkage to the Medical Birth Registry of Norway. All maternity units in Norway must notify births to the Medical Birth Registry of Norway. In the NINFEA study cohort, CHDs were reported in the second questionnaire, compiled 6 months after birth. Mothers compiled a checklist that included prespecified anomalies. If the child died or had any surgery performed in the first 6 months, the cause of death and type of surgery were also checked to see if any CA was reported. Data were coded using *ICD‐10* codes by an experienced pediatrician and were reassessed by an independent physician. Further details of the sources of data for CHDs in each cohort are provided in Data S3.

In all studies, our main outcome was any CHD. Where data allowed (ie, when we had full *ICD‐10* codes), any CHD was defined according to European Surveillance of Congenital Anomalies, which excludes isolated patent ductus arteriosus and peripheral pulmonary artery stenosis in preterm births (gestational age, <37 weeks) (Table S2). We also categorized cases into severe CHD (heterotaxia, conotruncal defect, atrioventricular septal defect, anomalous pulmonary venous return, left ventricle outflow tract obstruction, right ventricle outflow tract obstruction, or other complex defects) and the remainder as nonsevere CHD (patent ductus arteriosus [in full‐term infants], valvular pulmonary stenosis, ventricular septal defect, atrial septal defects, unspecified septal defects, isolated valve defects, other specified heart defects, or unspecified heart defects)^33^, ^34^ (Table S2).

### Confounders

Analyses were adjusted for several confounders based on their known or plausible influence on ≥1 of the maternal pregnancy exposures and on CHD: maternal age (all exposures), parity (all exposures), ethnicity (all exposures), socioeconomic position (all exposures), smoking (for BMI and alcohol analyses), and alcohol use (for BMI and smoking analyses). In the paternal negative control analyses, confounders were similar: fathers' age (all exposures), number of children (all exposures), ethnicity (all exposures), socioeconomic position (all exposures), smoking (for BMI and alcohol), and alcohol use (for BMI and smoking). We also adjusted for offspring sex in all adjusted analyses. We used educational attainment for both parents' measures of socioeconomic position. Full details of our selection and harmonization of confounders are provided in Data S4.

### Statistical Analysis

Analyses were conducted in either R (version 3.6.1) or Stata (version 16). An analysis plan was written and published in October 2019, with any subsequent changes and their rationale documented in the publication.^35^ All associations between exposures and CHDs were performed within participating studies using logistic regression (binary for main analyses and multinomial for CHD severity analyses). In the 2 largest cohorts (DNBC study and MoBa), we assessed deviation from linearity in our models in the BMI analyses by running our main confounder‐adjusted model with BMI split into fifths. We ran regression models with these fifths as 4 indicator variables (nonlinear) and compared this model with one in which the fifths were treated as a continuous (score) variable. We used a likelihood ratio comparison to compare these 2 models. All analyses were run (1) unadjusted; (2) adjusted for maternal/paternal age, socioeconomic position, parity, ethnicity, smoking and/or alcohol (depending on exposure), and offspring sex; and (3) adjusted for all confounders (as in 2) as well as the other parent's exposure. In the adjusted models, studies were asked to adjust for as many of the confounders as possible. All analyses were performed with maximal numbers (ie, numbers included in each model will vary because of missing data on exposure/outcome or confounders). In a sensitivity analysis, we repeated our main analyses using complete‐case data to assess whether missing data were influencing the results.

For the main negative control analyses (ie, where we directly compared maternal with paternal exposure‐CHD associations), we used multivariable logistic regression in which both maternal and paternal exposures were adjusted for the other parent's exposure (model 3 above). This produces a maternal association that adjusts for maternal confounders as well as the paternal exposure, and similarly a paternal association adjusting for paternal confounders and the maternal exposure. The rationale for mutually adjusting for the other parent's exposure is that parental BMI, smoking, and alcohol may relate to each other through assortative mating and/or convergence of behaviors that occurs over time in couples.^36^ Causal structural graphs together with simulated data show failure to undertake this mutual adjustment will bias the negative control analysis results.^37^ Also, paternal exposures may have some intrauterine impact (eg, via passive smoking or paternal support for the mother to reduce alcohol and have a normal BMI during her preconceptual period or in pregnancy).^38^ Mutual adjustment for maternal and paternal confounders was necessary for ensuring both parental results were fully adjusted. Comparisons between maternal and paternal associations from this model were assessed by visually comparing the 2 results. In addition, statistical evidence of any differences was obtained by calculating differences in log odds of CHD between the fathers' and mothers' associations and report of the corresponding *P* value (*P*_difference_), under the null hypothesis that there is no difference between the maternal and paternal estimate.

Analyses were conducted separately in each study and then meta‐analysed using the *meta* package in R.^39^ All the data used in the present study originated from European birth cohorts, with broadly similar methods, and therefore, we assumed that they were each estimating an association from the same underlying populations and used a fixed‐effects meta‐analysis. To explore this assumption, differences between studies were assessed using I^2^ and Cochrane Q *P* values for heterogeneity.^40^

### Additional Analyses

We repeated the main and subgroup (by CHD severity) analyses after excluding infants with any known chromosomal/genetic or maternal drug defects. Methods of data collection and definition of these variables can be found in Table S3. We also repeated analyses in mothers only including those with smoking data in the first trimester. Folic acid supplementation has been shown to lower risk of birth defects and adverse pregnancy outcomes.^41^, ^42^ We repeated the adjusted maternal analyses with additional adjustment for first‐trimester folic acid supplementation (yes/no).

## Results

### Participant Characteristics

Figures S1 through S7 show flowcharts designating the assignment of participants into analysis groups for each cohort. In total, 7 cohorts, including 232 390 offspring with 2469 CHD cases (1.1%), were included. The prevalence of CHD was close to 1% in most cohorts, with the lowest being in ABCD (0.4%) and the highest in DNBC study (1.4%) (Table). The Table shows the distributions of maternal and paternal characteristics for each cohort. Mean maternal age across the cohorts was broadly similar (all late 20s to early 30s). Mean BMI was also similar across the cohorts, but proportions in different categories varied, with the lowest prevalence of prepregnancy/early‐pregnancy obesity seen in NINFEA study (5%) and the highest in BiB study (21%). There was also variation in maternal smoking and alcohol consumption across the cohorts, with notably high levels of both smoking (25% and 26%) and alcohol (55% and 45%) in ALSPAC and DNBC study, respectively. Fathers were generally older than mothers and more likely to smoke and drink alcohol, with the overall patterns of between‐study differences being similar to those for the mothers. There were differing levels of missing data in each cohort (summarized in Table S4 and also illustrated in cohort‐specific flow charts [Figures S1 through S7]). To check whether missing data influenced any of our results, we report complete‐case analysis results for our main analyses in the Supplementary Material. Overall, complete‐case results from meta‐analyses were comparable (Tables S5 through S8). Below, we present our main results separated by exposure. We include supplementary results for BMI (Figures S8 through S20, Tables S9 and S10), smoking (Figures S21 through S27), and alcohol (Figures S28 through S32 and Table S11) analyses in the Supplementary Material.

### BMI and CHDs

**Figure 1:**
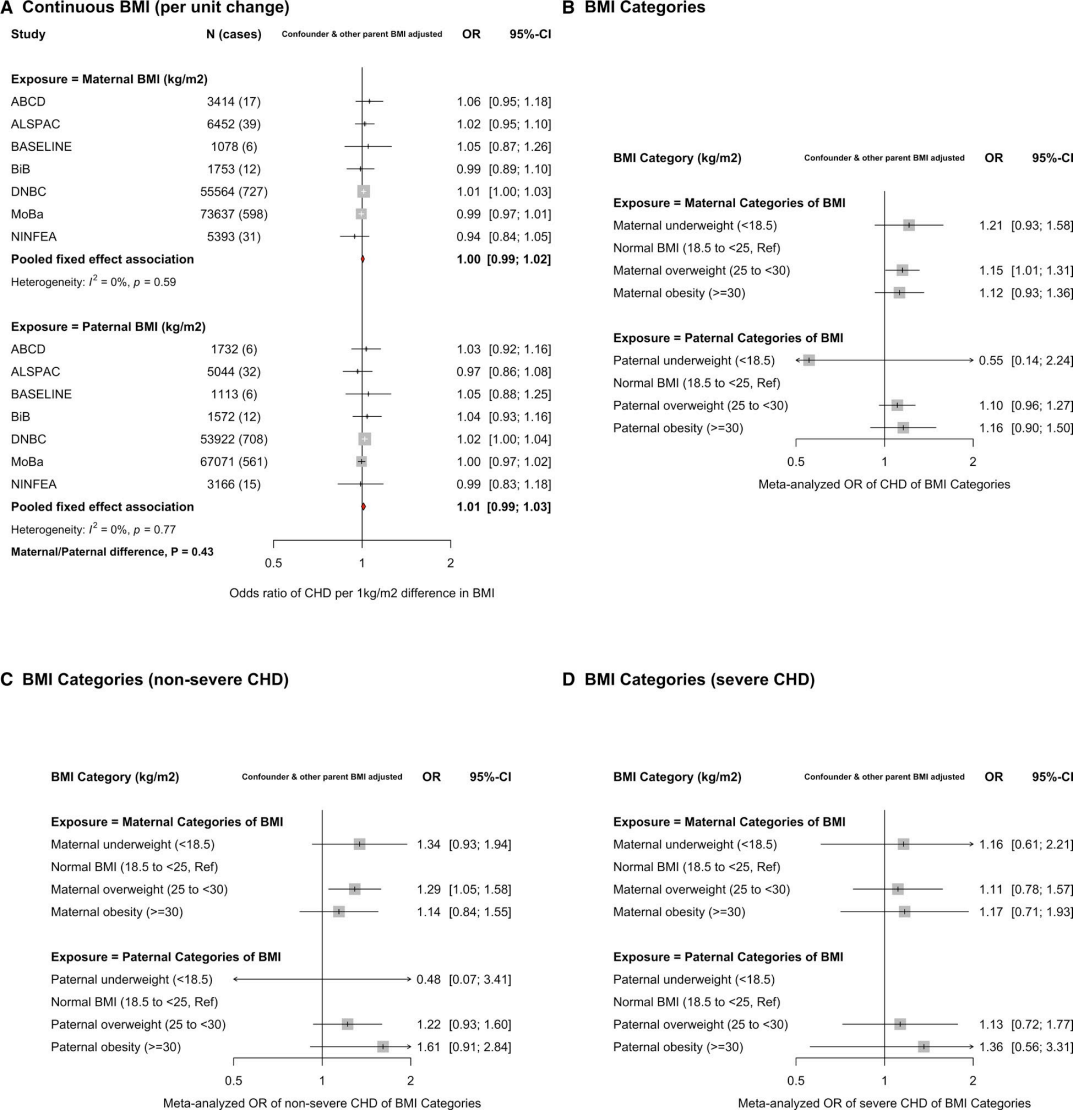
Associations between maternal and paternal prepregnancy/early‐pregnancy body mass index (BMI) and offspring congenital heart disease (CHD). **A**, Odds ratios (ORs) of CHD for a 1‐unit (1‐kg/m^2^) difference in maternal BMI (top) and paternal BMI (bottom) in each study and pooled across studies. **B**, The pooled (across ALSPAC [Avon Longitudinal Study of Parents and Children], BiB [Born in Bradford] study, DNBC [Danish National Birth Cohort] study, and MoBa [Norwegian Mother, Father and Child Cohort Study]) results for maternal (top) and paternal (bottom) BMI categories. Results are ORs of CHD in comparison to normal BMI. **C** and **D**, ORs of nonsevere CHD and severe CHD, respectively, for BMI categories in comparison to normal BMI (pooled across ALSPAC, BiB study, DNBC study, and MoBa). All results are adjusted for confounders (depending on cohort: maternal and paternal age, education, ethnicity, smoking, alcohol, maternal parity, and offspring sex) as well as the other parent's BMI. The study‐specific results for BMI categories are shown in Figures S15 through S20. In **D**, there were too few cases with paternal BMI data to report results. These analyses are from the LifeCycle project, a consortium that brings birth cohorts together and harmonizes individual‐level data for their use in research.^13^ All LifeCycle studies with eligible data were included in this study. More information on each can be found as follows: ABCD (The Amsterdam Born Children and Their Development Study),^15^ ALSPAC,^16^, ^17^ BASELINE (Cork Scope Baseline Study),^18^ BiB study,^19^ DNBC study,^20^ MoBa,^21^, ^22^ and NINFEA (Nascita e Infanzia: gli Effetti dell'Ambiente [Birth and Childhood: Effects of the Environment]) study.^23^, ^24^

In confounder and other parent BMI‐adjusted analyses, there was no difference in the odds of offspring CHD per 1‐kg/m^2^ difference in maternal BMI (odds ratio [OR], 1.00; 95% CI, 0.99–1.02) or paternal mean BMI (OR, 1.01; 95% CI, 0.99–1.03) (*P*_difference_=0.43), with both being close to the null (Figure 1A). Unadjusted and confounder‐only adjusted results did not differ notably from those presented in Figure 1 (Figure S8). The odds of CHD did not clearly increase linearly in mothers or fathers in DNBC study or MoBa (Figures S9 and S10). Analyses of continuously measured BMI with CHD cases separated into nonsevere and severe showed similar null associations for both mothers and fathers (Figure S11).

In analyses of BMI categories, there were increased odds of offspring CHD in overweight and obese mothers and fathers compared with those of a normal BMI, with similar magnitudes of association in both parents (*P*_difference_ overweight=0.65 and *P*_difference_ obese=0.83) (Figure 1B). Underweight mothers had an increased odds of offspring CHD, whereas underweight fathers had a decreased odds of offspring CHD. Because of small numbers of underweight parents, particularly fathers, however, results were imprecise, with wide CIs, and there was no statistical evidence for between parental differences for underweight (*P*_difference_ underweight=0.27). Individual study results for BMI categories are shown in Figures S15 through S17. Positive parental associations of overweight and obesity were also observed for both nonsevere (Figure 1C) and severe (Figure 1D) CHDs, with similar magnitudes of association in mothers and fathers. Individual study results for BMI categories and CHD severity are shown in Figures S18 through S20.

### Smoking and CHDs

**Figure 2:**
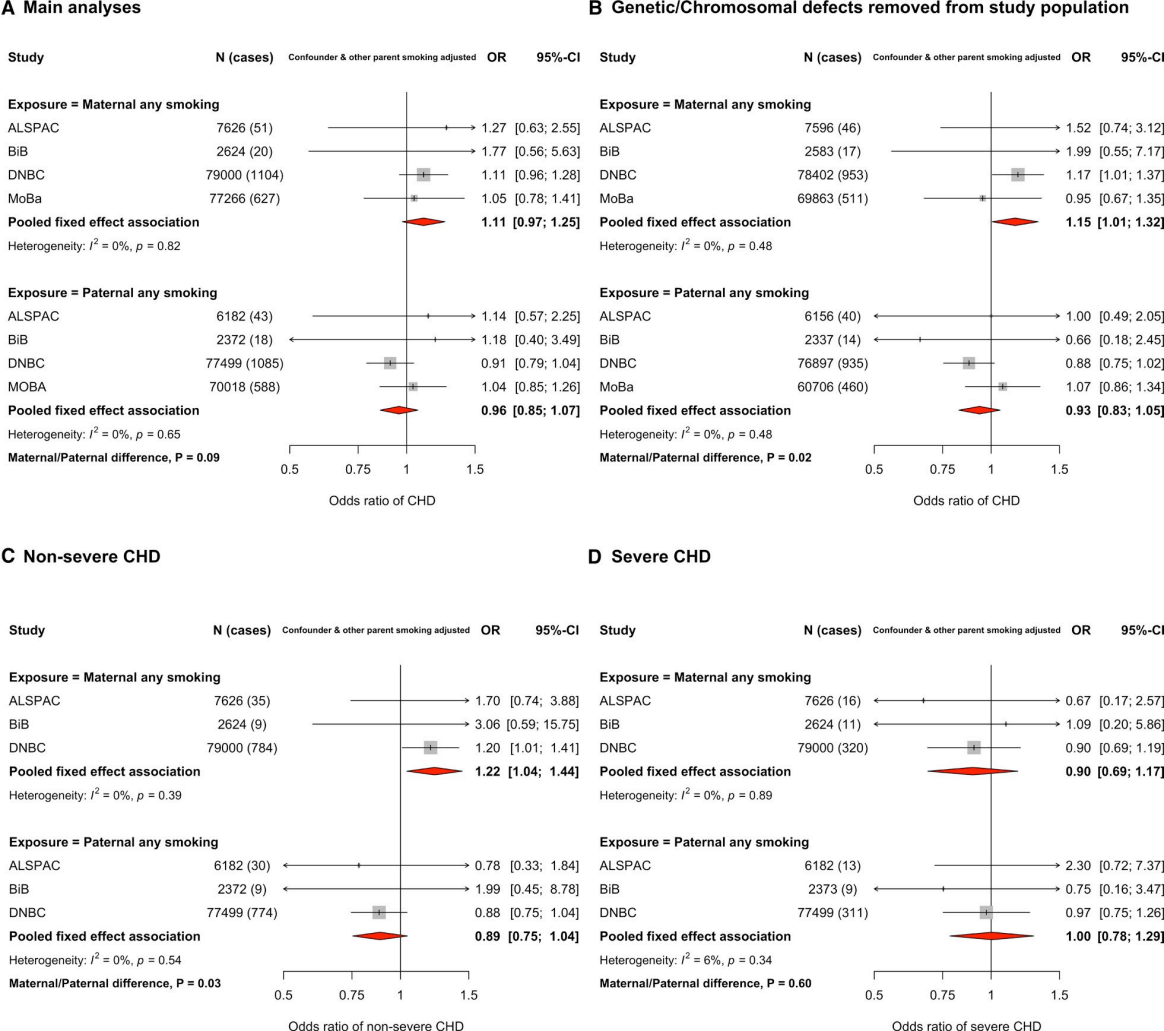
Associations in each study and pooled across studies for maternal and paternal pregnancy smoking and offspring congenital heart disease (CHD). Maternal first‐trimester smoking was prioritized and used where possible. **A**, Odds ratios (ORs) of any CHD for maternal smoking during pregnancy (top) and paternal smoking (bottom). **B**, ORs of any CHD after removing those with a chromosomal/genetic defect from the study population. **C** and **D**, ORs of nonsevere CHD and severe CHD, respectively. All results are adjusted for confounders (depending on cohort: maternal and paternal age, education, ethnicity, alcohol, maternal parity, and offspring sex) as well as the other parent's smoking. These analyses are from the LifeCycle project, a consortium that brings birth cohorts together and harmonizes individual‐level data for their use in research.^13^ All LifeCycle studies with eligible data were included in this study. More information on each can be found as follows: ABCD (The Amsterdam Born Children and Their Development Study),^15^ ALSPAC (Avon Longitudinal Study of Parents and Children),^16^, ^17^ BASELINE (Cork Scope Baseline Study),^18^ BiB (Born in Bradford) study,^19^ DNBC (Danish National Birth Cohort) study,^20^ MoBa (Norwegian Mother, Father and Child Cohort Study),^21^, ^22^ and NINFEA (Nascita e Infanzia: gli Effetti dell'Ambiente [Birth and Childhood: Effects of the Environment]) study.^23^, ^24^

In confounder and other parental smoking‐adjusted analyses, maternal smoking in pregnancy was associated with increased odds of CHD (OR, 1.11; 95% CI, 0.97–1.25), whereas paternal smoking at the time of their partners pregnancy did not increase odds of offspring CHD (OR, 0.96; 95% CI, 0.85–1.07) (*P*_difference_=0.09) (Figure 2A). When removing offspring with a chromosomal/genetic defect, there was stronger statistical evidence of a difference between maternal and paternal smoking (*P*_difference_=0.02) (Figure 2B). Results for unadjusted analyses were consistent with the confounder and mutual parent smoking‐adjusted result, whereas confounder‐only analyses were slightly attenuated for maternal smoking (Figure S21). Maternal smoking results were similar when analyses were restricted to studies with confirmed first‐trimester smoking (Figure S22). A positive association between maternal smoking and offspring CHD was also seen with nonsevere CHDs (OR, 1.22; 95% CI, 1.04–1.44), although not with severe CHDs (OR, 0.90; 95% CI, 0.69–1.17) (Figure 2C and 2D and Figure S23). When we analyzed maternal smoking frequency categories (ie, none, light, and heavy smoking), the results did not support an effect of heaviness over and above what we saw with any smoking (Figure S24). The maternal and paternal associations for these categories were statistically consistent (*P*_difference_=0.25 and *P*_difference_=0.38 for light and heavy smoking, respectively).

### Alcohol and CHDs

Because of lack of relevant paternal data, we were unable to undertake negative control analyses for any first‐trimester alcohol consumption. Maternal‐only associations for that exposure are presented herein, followed by the negative control analyses for levels of alcohol intake at any time in pregnancy. With adjustment for all confounders, any maternal first‐trimester alcohol consumption was not associated with odds of offspring CHD in meta‐analyses from 5 cohorts (OR, 1.03; 95% CI, 0.94–1.13) (Figure S28). There was a small increase in risk when restricting these analyses to nonsevere CHD (OR, 1.07; 95% CI, 0.93–1.22), although CIs included the null. Associations for severe CHD were null (OR, 0.91; 95% CI, 0.73–1.12) (Figure S29).

**Figure 3:**
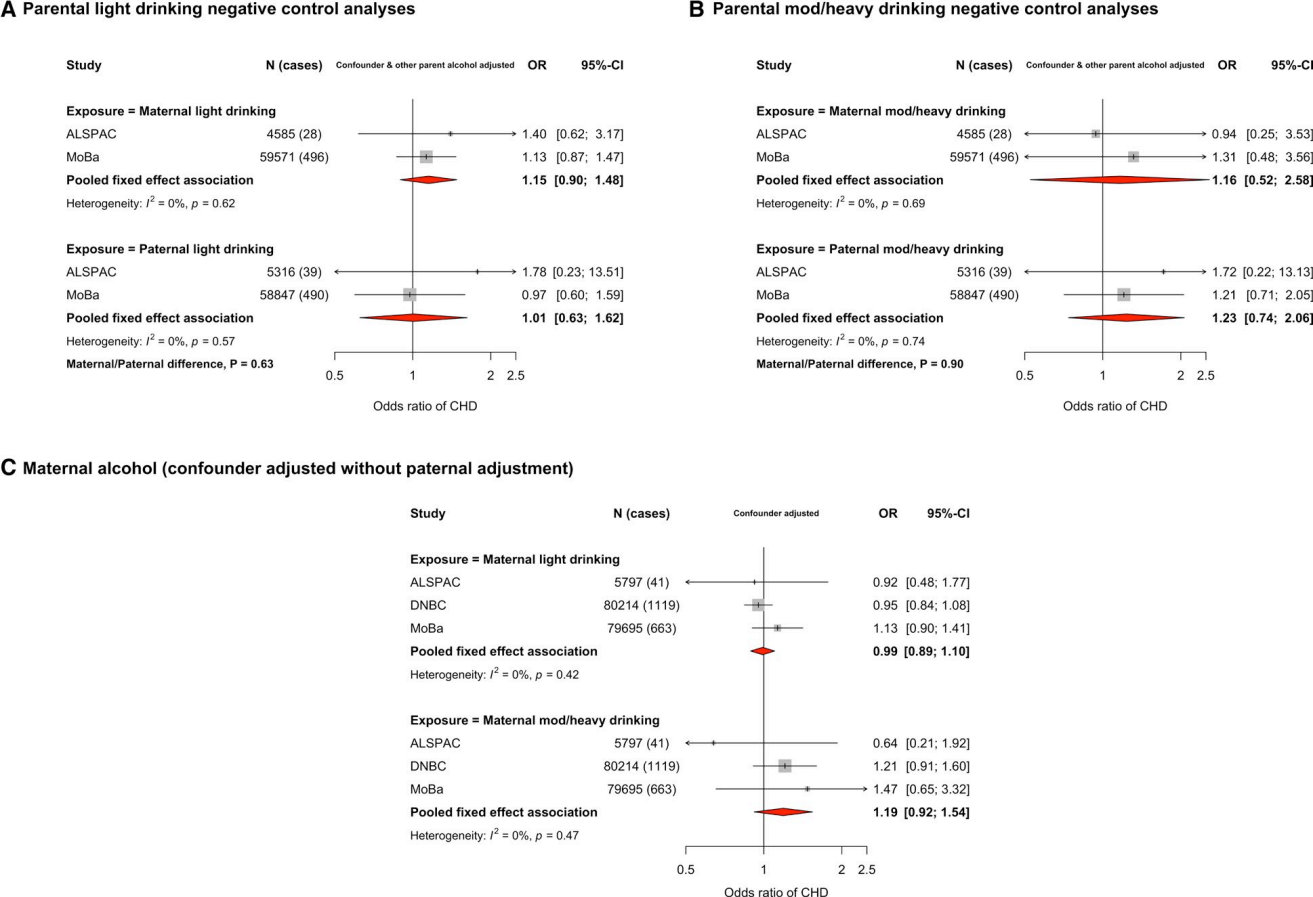
Associations in each study and pooled across studies for maternal and paternal pregnancy alcohol intake and any offspring congenital heart disease (CHD). **A**, Confounder and other parent's alcohol adjusted odds ratios (ORs) of any CHD for maternal light drinking during pregnancy (top) and paternal light drinking (bottom). **B**, Confounder and other parent's alcohol‐adjusted ORs of any CHD for maternal moderate/heavy drinking during pregnancy (top) and paternal moderate/heavy drinking (bottom). **C**, Confounder‐adjusted ORs of any CHD for maternal light drinking during pregnancy (top) and maternal moderate/heavy drinking (bottom). Confounders (depending on cohort): maternal and paternal age, education, ethnicity, smoking, maternal parity, and offspring sex (and other parental alcohol intake in **A** and **B**). Definitions for maternal/paternal alcohol intake are described in the Methods section. These analyses are from the LifeCycle project, a consortium that brings birth cohorts together and harmonizes individual‐level data for their use in research.^13^ All LifeCycle studies with eligible data were included in this study. More information on each can be found as follows: ABCD (The Amsterdam Born Children and Their Development Study),^15^ ALSPAC (Avon Longitudinal Study of Parents and Children),^16^, ^17^ BASELINE (Cork Scope Baseline Study),^18^ BiB (Born in Bradford) study,^19^ DNBC (Danish National Birth Cohort) study,^20^ MoBa (Norwegian Mother, Father and Child Cohort Study),^21^, ^22^ and NINFEA (Nascita e Infanzia: gli Effetti dell'Ambiente [Birth and Childhood: Effects of the Environment]) study.^23^, ^24^

In confounder and other parental alcohol‐adjusted analyses, there was weak evidence of an association between maternal light alcohol intake and CHDs (OR, 1.15; 95% CI, 0.90–1.48), which appeared stronger than that seen for paternal alcohol (OR, 1.01; 95% CI, 0.63–1.62), although with no strong statistical support for a difference (*P*_difference_=0.63). Associations for moderate/heavy intake were consistent for maternal and paternal alcohol (*P*_difference_=0.90), with point estimates showing weak positive associations, but with wide CIs that included the null (Figure 3A and 3B). We did not test associations between levels of alcohol intake and CHD severity because of small numbers. Because of the small number of cohorts having paternal alcohol data, we also show confounder‐adjusted models (without mutual paternal adjustment) for maternal alcohol intake (Figure 3C). The point estimate for maternal light drinking was close to the null and that for heavy drinking suggested it resulted in increased risk of offspring CHD. However, both of these estimates had wide CIs because of relatively few women reporting drinking (particularly heavily) during pregnancy. Results in unadjusted analyses were unchanged (Figure S30).

### Between‐Study Heterogeneity and Additional Analyses

We have included heterogeneity statistics (I^2^ and *P*_heterogenity_) in all figures. Analyses of continuously measured BMI and severe CHDs in additional analyses (Figure S14) and BMI analyzed as categories with severe CHDs (Figures S19 and S20) were the only results where we found any statistical evidence of heterogeneity. Across the remaining analyses for all exposures, there was no strong evidence of between‐study heterogeneity. Removal of those with any known genetic/chromosomal defects from the study population did not notably alter any main or severity subgroup analyses for BMI and alcohol consumption. However, for smoking, removal of offspring with a chromosomal/genetic defect increased the magnitude of the association for maternal smoking and CHDs (OR, 1.15; 95% CI, 1.01–1.32), and slightly decreased that for paternal smoking (OR, 0.93; 95% CI, 0.83–1.05) (*P*_difference_=0.02) (Figure 2B). Furthermore, the positive association between maternal smoking and nonsevere CHDs was slightly stronger when removing those with chromosomal/genetic defects from the study population (OR, 1.25; 95% CI, 1.05–1.49) (Figure S26). All maternal results were materially unchanged after additional adjustment for folic acid supplementation (Figures S12, S27, and S32).

## Discussion

In this large multicohort study, we found evidence that maternal pregnancy smoking may increase offspring CHD risk via intrauterine mechanisms and that this may be driven by a specific effect on nonsevere CHDs. We did not find robust evidence to suggest a causal intrauterine effect of higher maternal prepregnancy/early‐pregnancy mean BMI or overweight or obesity on offspring CHD risk. Nor did we find evidence of an intrauterine effect of alcohol consumption on offspring CHD risk, although we acknowledge that for alcohol, we had less data and limited statistical power. To our knowledge, this is the first study to use a parental negative control method to explore whether maternal exposures have a causal intrauterine effect on offspring CHDs or whether associations are explained by residual confounding, which would generate a similar association for parental exposures.

We found increased odds of offspring CHD in mothers who were overweight and obese. This is consistent with the most recent systematic review and meta‐analysis, which included 2 416 546 participants (57 172 with offspring CHD) from 19 studies and reported increased risk of any offspring CHD in women who were overweight or obese during pregnancy.^5^ However, adjustment for confounders was poor, with 10 of the 19 included studies not providing information on confounder adjustment or not adjusting for any confounders. With more stringent confounder adjustment and the findings from a negative control study, our results suggest that the increased risk of offspring CHD in overweight and obese mothers is largely the result of residual confounding. We also found that mothers who were underweight at the start of pregnancy were at increased risk of having offspring with CHD, whereas underweight in fathers appeared to be protective of offspring CHD. There were 9537 underweight mothers (4.4%) but only 680 underweight fathers (0.4%) in our study population, making the paternal analyses imprecise and our negative control analyses lacking in power to reliably identify parental differences. The recent systematic review mentioned above did not report on associations of underweight with CHDs because too few studies looked at this.

A large Swedish linkage study of >2 million singleton live born infants (born between 1992 and 2012, with 28 628 CHD cases) has explored associations with maternal underweight, as well as overweight and 3 grades of obesity.^7^ It is difficult to directly compare the results from that study with ours as we only present results for any CHD (and CHD stratified by severity), whereas they only present associations of maternal BMI with specific subtypes of CHDs. The fact that we lack statistical power in our study to explore associations with specific subtypes is a limitation. However, magnitudes of associations of BMI categories and nonsevere CHDs in our study appear to be broadly consistent with several nonsevere defects in the large Swedish study, including atrial septal defects and isolated valve defects. In their study, risks of offspring CHD were similar in underweight compared with normal weight women for all types of CHD (analyzed individually), except for mitral to tricuspid valve defects (14 cases), pulmonary valve defects (24 cases), and right ventricular defects (5 cases), where there was some evidence of increased prevalence with underweight. However, these estimates were based on small numbers and hence imprecise, with CIs including the null. Although our findings suggest maternal underweight might increase offspring risk of CHDs, we lacked power to rule out residual confounding in our negative control analyses, and as noted above the large Swedish study had limited power to determine precise effects in relation to maternal underweight for specific types of CHD where point estimates suggested potentially important magnitudes of increased risk. Other studies that we are aware of have not explored associations of maternal underweight. Thus, any possible effect of maternal underweight on CHD risk remains unclear. As the prevalence of CHD in some low‐ and middle‐income countries is high,^43^ and these countries currently experience the double burden of undernutrition and overnutrition, we would argue that further exploration of any possible impact of maternal underweight is warranted.

Consistent with our findings, a recent meta‐analysis of >8 million participants (137 575 CHD cases) from 125 studies reported positive associations between maternal pregnancy smoking and offspring CHDs.^8^ There was substantial heterogeneity (I^2^=89%) in their pooled results, and only 68% of the included studies report adjustment for confounders. The authors also report positive associations between maternal passive smoking and paternal active smoking with offspring CHDs, both of which (somewhat unexpectedly) had stronger magnitudes of association than results from maternal active smoking. Our results, including the negative control study, add to the previous research findings by providing more robust evidence that these associations for maternal smoking are unlikely to be explained by residual confounding and are potentially causal. Other research has shown that pregnancy smoking is a risk factor for orofacial clefts.^44^ The prevalence of CHD is around 1% in the general population, as shown in our study, yet in those with orofacial clefts, CHD prevalence rates of up to 20% have been reported.^45^ Both the heart and the palate develop during early pregnancy, around weeks 5 to 9. Therefore, it is plausible that smoking in early pregnancy could disturb common biological pathways in these conditions. We found that the associations for maternal smoking were possibly largely driven by an effect in nonsevere CHDs, with the association strengthening when those with chromosomal or genetic defects were removed. Previous research has reported positive associations between maternal smoking and septal defects, in particular for atrial septal defects,^46^, ^47^, ^48^ which are defined as nonsevere according to the classification system used in our study. However, caution is needed in interpreting results by subgroups based on severity. First, one of the largest studies (MoBa) did not have information on case severity and so the severity subgroup analyses are based on different participants and have lower statistical power than in the main analyses. Second, even had all studies been included in the severity analyses, by definition, subgroup analyses have limited power in comparison to main analyses. Third, and more important, caution is required with any subgroup analyses as it is common for multiple characteristics to differ between subgroups in addition to the subgroup defining feature (herein, CHD severity).

In confounder‐adjusted analyses, maternal alcohol consumption in the first trimester of pregnancy was not associated with offspring CHD. There was some evidence that maternal moderate or heavy alcohol consumption any time in pregnancy was associated with increased risk of offspring CHD. Although associations between mothers and fathers light, moderate, and heavy alcohol consumption, compared with none, were statistically consistent, only 2 cohorts (80 627 participants, 703 with offspring CHD) had alcohol information on fathers around the time of their partners pregnancy. Associations for fathers in particular were imprecise, with wide CIs. Two recent meta‐analyses found consistent modest increases in risk of offspring CHD in mothers reporting alcohol consumption in pregnancy (OR, 1.11 [95% CI, 0.96–1.29]^49^ and OR, 1.16 [95% CI, 1.05–1.27]^9^). Although the first of these concluded “no association,” it can be seen that the results for the 2 are consistent, and the larger sample size of the second has increased precision. Of note, the second of these studies also explored paternal consumption and found increased risk of offspring CHD related to fathers' alcohol consumption (OR, 1.44 [95% CI, 1.19–1.74]).^9^ Although the OR for fathers' consumption suggests a stronger effect, the CIs are wide, and the result is statistically consistent with that for mothers' alcohol consumption. As in our study, there were fewer studies with data on paternal alcohol consumption around the time of their partners pregnancy. Taken together with our findings, these suggest that positive associations of maternal alcohol consumption with offspring CHD may be attributable to residual confounding rather than a causal intrauterine effect.

The key strengths of this study are its large sample size, the use of a negative paternal exposures control study, and the pooling of results from several cohort studies that are less prone to selection bias that can occur in case‐control studies and are not selected on the basis of publication, but on being part of an existing collaboration. The latter reduces the risk of publication bias as studies were included if they had data and not on the basis of (published) results. This also allowed us to explore replication across studies, and the consistency of findings between studies in our main analyses adds confidence to our conclusions.

The use of harmonized data from LifeCycle is a strength that limits between‐study heterogeneity. However, harmonizing data across several studies, as we have done in LifeCycle, can mean that some variables lose detail. Herein, that is particularly relevant for the exposure and confounding variables. For example, we were not able to explore pack weeks of smoking across the entire pregnancy. Simplified confounder measurements, such as Western versus non‐Western for ethnicity, could result in residual confounding if more specific ethnic groups have strong influences on exposure and outcome. Furthermore, there were other confounders that we considered, including type 1/pre‐existing diabetes mellitus and physical activity, but had too few numbers (diabetes mellitus) across all cohorts or too few studies with data (physical activity) to include. However, we aimed to address any form of residual confounding in our paternal negative control analyses. Under the assumption that adjusted for but poorly measured (eg, ethnicity) or unadjusted for (eg, physical activity) confounders influence paternal exposures in the same direction and to the same extent as in mothers, observing parental consistency of association implies that the maternal association is influenced by residual confounding.

We were not able to fully harmonize outcome data, with the key differences between studies being the extent to which they only included cases that were diagnosed antenatally or at birth or whether they included cases later in life. MoBa (N=101 975 participants and N=879 cases) only had cases diagnosed antenatally or around the time of birth, with the remaining cohorts having diagnoses beyond antenatal care, ranging from 6 months to 25 years. Many previous studies have only included cases diagnosed at birth or early infancy. They, and the cohorts included herein that only have these early life cases, may be biased by outcome misclassification (ie, the offspring who would have been diagnosed later in life are treated as not having CHD). This is an important point for consideration because although most CHDs are identified in utero or at birth, many are diagnosed after discharge from hospital during childhood or even adulthood.^50^ Therefore, it is reassuring that our main results are largely consistent across studies. In confounder and other parent‐adjusted smoking analyses, the weakest association was found in the MoBa cohort. It is likely that we missed some nonsevere cases in MoBa, which were diagnosed later in life. Given that we demonstrate the smoking results were largely driven by nonsevere CHDs, this could have biased MoBa (and therefore meta‐analysis) results toward the null.

The negative control analyses assume that factors that would confound the maternal exposure‐offspring CHD associations would have a similar magnitude and direction of confounding for the equivalent paternal associations, irrespective of whether the confounders are measured or if measured how accurately and precisely they are measured. This is likely to be true for paternal negative control exposure studies, as used herein.^10^, ^11^ Both maternal and paternal BMI, smoking, and alcohol consumption could have preconceptual effects via influences on gametes, including epigenetic changes. Any such effects would plausibly differ between mothers and fathers, and for the mother would be in addition to potential intrauterine effects, such that we may still expect stronger maternal associations. Furthermore, there is little conclusive evidence of effects of factors, such as smoking, on gametes that do not render them infertile but are sufficient to influence embryo development and hence CHDs, as such studies are difficult in humans. Heart development occurs in utero (specifically in early pregnancy), and we would expect passive paternal smoke inhalation to expose the fetus to a lower level of exposure than active maternal smoking. As proof of concept, paternal smoking does not associate with offspring birth weight or fetal growth parameters (assessed by repeated ultrasound), in contrast to maternal smoking, which has marked effects.^12^ It is possible that potential differences in misreporting smoking and alcohol consumption between mothers and fathers could produce spurious parental differences. Pregnant women are likely to underreport whether they smoke or drink alcohol and the amount they smoke or drink, because of the social stigma of these, particularly in recent decades. As the report of alcohol and smoking in the LifeCycle cohorts was collected early in pregnancy, it is likely to be random in relation to an offspring CHD as the vast majority would not have been diagnosed. Hence, this underreporting would be expected to attenuate any true effect of smoking/alcohol on CHD toward the null. This misclassification is less likely in fathers. Thus, the specific positive association of maternal smoking on CHDs and its difference to the paternal association may be underestimated.

Finally, only 47% of mothers with smoking data in our study had this specifically during the first trimester. Paternal smoking was defined as smoking around the time of pregnancy, with no specific trimester measurements. However, although amount smoked may change across pregnancy, it is highly likely that any smoking in later trimesters is a strong proxy for smoking in the first trimester. More important, we have shown that our results using only maternal first‐trimester smoking are consistent with our main results. Similarly, paternal smoking at any time during pregnancy is likely to be a good proxy for smoking in early pregnancy. However, we acknowledge it would be useful to have more detailed data on both parents across all trimesters to explore whether association magnitudes vary by trimester.

In summary, we found evidence to support a causal intrauterine effect of maternal smoking on any CHD, particularly with nonsevere CHDs, but did not find robust evidence for a causal effect of maternal BMI or alcohol on offspring CHD risk. Although everyone should be encouraged not to smoke, and all clinical guidelines advocate not starting smoking, and if women do smoke, to quit before becoming pregnant, there are still high rates of smoking in some groups, particularly those from deprived backgrounds. In the studies included in this article, in 2 contemporary cohorts, BASELINE (Ireland), with births occurring between 2008 and 2011, and the BiB cohort (United Kingdom), with births occurring between 2007 and 2011, smoking prevalence rates were 25% and 16%, respectively. The prevalence in the BiB cohort masks the high rate in White British women (33%) who are from socioeconomically deprived backgrounds, as >50% of births in that cohort are to Pakistani women who have low rates of smoking (3%).^19^ It is possible that emphasizing the potential adverse effect on CHDs in specific groups might help in supporting women of reproductive age not to start smoking and women who are smoking at the start of pregnancy to be encouraged to quit. Furthermore, understanding the specific mechanisms that link maternal smoking to increased offspring CHD risk could identify targets for interventions for its prevention.

## Sources of Funding

The LifeCycle project received funding from the 452 European Union's Horizon 2020 Research and Innovation Programme 453 (Grant Agreement No. 733206 LifeCycle). This study is also supported by the British Heart Foundation (AA/18/7/34219), Bristol National Institute for Health Research (NIHR) Biomedical Research Centre grant, European Research Council (Advanced Grant; 669545), and US National Institutes of Health (R01 DK10324). K. Taylor is supported by a British Heart Foundation Doctoral Training Program (FS/17/60/33474). K. Taylor, Dr Elhakeem, and Prof Lawlor work in a unit that is supported by the University of Bristol and UK Medical Research Council (MC_UU_00011/6). Prof Lawlor is supported by a British Heart Foundation Chair in Cardiovascular Science and Clinical Epidemiology (CH/F/20/90003) and a NIHR Senior Investigator (NF‐0616‐10102). Prof Caputo is supported by the British Heart Foundation Chair in Congenital Heart Disease (CH/1/32804). Dr Pinot de Moira is funded by a Lundbeck Foundation Fellowship (R264‐2017‐3099). Work by Dr Harris is partly supported by the Research Council of Norway through its Centers of Excellence Funding Scheme, project No. 262700. Details of funding for each individual LifeCycle cohort that has contributed to this study are provided below. ABCD (The Amsterdam Born Children and Their Development Study) was supported by the Netherlands Organization for Health Research and Development (grant 2100.0076) and the Electromagnetic Fields and Health Research Program (grants 85600004 and 85800001). Core funding for ALSPAC (Avon Longitudinal Study of Parents and Children) is provided by the UK Medical Research Council and Wellcome (217065/Z/19/) and the University of Bristol. Many grants have supported different data collections, including for some of the data used in this publication, and a comprehensive list of grant funding is available on the ALSPAC website (http://www.bristol.ac.uk/alspac/external/documents/grant‐acknowledgements.pdf). The BASELINE (Cork Scope Baseline Study) Birth Cohort Study was funded by the National Children's Research Centre (Project grant: Jan 2009). BiB (Born in Bradford) study has received core support from Wellcome Trust (WT101597MA), a joint grant from the UK Medical Research Council and UK Economic and Social Science Research Council (MR/N024397/1), the British Heart Foundation (CS/16/4/32482), and the NIHR Applied Research Collaboration Yorkshire and Humber (NIHR200166) and Clinical Research Network. The views expressed in this publication are those of the author(s) and not necessarily those of the National Institute for Health Research or the Department of Health and Social Care. The DNBC (Danish National Birth Cohort) study was supported by the Danish Epidemiology Science Centre, the Lundbeck Foundation (grant 195/04), the Egmont Foundation, the March of Dimes Birth Defect Foundation, the Augustinus Foundation, and the Medical Research Council (grant SSVF 0646). The MoBa (Norwegian Mother, Father and Child Cohort Study) is supported by the Norwegian Ministry of Health and Care Services and the Ministry of Education and Research. The NINFEA (Nascita e Infanzia: gli Effetti dell'Ambiente [Birth and Childhood: Effects of the Environment]) study was partially funded by the Compagnia San Paolo Foundation.

## Disclosures

Prof Lawlor reports support from Roche Diagnostics and Medtronic Ltd for research unrelated to that presented herein. The remaining authors have no disclosures to report.

**Table 1:** Characteristics of the Participating Cohorts

Characteristics	Category	ABCD (N=8131)	ALSPAC (N=13 049)	BASELINE (N=1436)	BiB Study (N=12 799)	DNBC Study (N=89 107)	MoBa (N=101 975)	NINFEA Study (N=5893)
Country		The Netherlands	United Kingdom	Ireland	United Kingdom	Denmark	Norway	Italy
Recruitment period		2003–2004	1991–1992	2008–2011	2007–2011	1996–2002	1999–2008	2005–2016
Offspring								
CHD	Any	34 (0.4)	103 (0.8)	10 (0.7)	145 (1.1)	1264 (1.4)	879 (0.9)	34 (0.6)
CHD severity in those with CHD	Nonsevere	…	73/103 (70.9)	…	93/145 (64.1)	896/1264 (70.9)	…	27/34 (79.4)
	Severe	…	30/103 (29.1)	…	52/145 (35.9)	368/1264 (29.1)	…	7/34 (20.6)
Chromosomal/genetic defects*		26 (0.3)	58 (0.4)	…	198 (1.5)	698 (0.8)	169 (0.2)	7 (0.1)
Maternal								
Age, y		30.7 (5.3)	28.9 (4.8)	30.7 (4.4)	26.0 (5.7)	29.9 (4.3)	30.2 (4.6)	33.1 (4.3)
BMI, kg/m^2^		23.1 (4.1)	22.6 (4.4)	24.4 (4.1)	26.0 (5.7)	23.6 (4.3)	24.0 (4.3)	22.5 (3.8)
BMI categories	Underweight (<18.5 kg/m^2^)	360 (4.9)	1271 (11.6)	23 (1.6)	444 (4.4)	3861 (4.5)	3077 (3.2)	501 (8.5)
	Normal (18.5–<25 kg/m^2^)	5270 (71.8)	7426 (67.7)	914 (63.6)	4586 (45.4)	57 894 (67.8)	63 706 (65.4)	4156 (70.5)
	Overweight (25–<30 kg/m^2^)	1245 (17.0)	1537 (14.0)	345 (24.0)	2952 (29.2)	16 578 (19.4)	21 280 (21.8)	826 (14.0)
	Obese (≥30 kg/m^2^)	467 (6.4)	736 (6.7)	154 (10.7)	2127 (21.0)	7017 (8.2)	9337 (9.6)	286 (4.9)
Pregnancy smoking	Yes^†^	769 (9.5)	3147 (24.7)^†^	357 (24.9)^†^	1788 (16.4)	22 514 (26.0)^†^	9650 (9.6)	472 (8.1)^†^
	Light	…	1684 (15.7)	…	1362 (12.5)	15 777 (17.9)	7856 (7.7)	438 (7.5)
	Heavy	…	1096 (10.2)	…	426 (3.9)	7431 (8.5)	1587 (1.6)	30 (0.5)
Pregnancy alcohol	Yes^†^	1686 (20.8)	6894 (54.6)^†^	527 (36.7)	…	38 733 (44.7)^†^	22 799 (27.7)^†^	1508 (25.8)^†^
	Light	…	3044 (46.8)	…	…	46 774 (52.9)	10 461 (12.4)	1416 (24.4)
	Moderat/heavy	…	871 (13.4)	…	…	3717 (4.2)	509 (0.6)	230 (3.9)
Parity	Nulliparous	4500 (55.3)	5645 (45.0)	1436 (100)	4912 (39.8)	42 203 (47.4)	46 988 (46.9)	4070 (72.4)
Education	Low	4035 (49.6)	2374 (20.0)	…	5717 (56.9)	22 225 (27.6)	2735 (2.9)	278 (4.8)
	Medium	2225 (27.4)	7985 (67.1)	208 (14.6)	1563 (15.6)	17 756 (22.0)	31 430 (33.1)	1892 (32.4)
	High	1871 (23.0)	1538 (12.9)	1219 (85.4)	2769 (27.6)	40 675 (50.4)	60 847 (64.0)	3677 (62.9)
Folic acid supplementation	Yes	5677 (70.7)	1070 (8.5)	…	…	56 998 (69.0)	74 466 (74.3)	4741 (82.5)
Paternal								
Age, y		35.1 (5.8)	30.9 (5.8)	32.2 (4.8)	30.4 (6.6)	32.2 (5.2)	32.7 (5.4)	36.2 (5.2)
BMI, kg/m^2^		25.0 (3.5)	25.2 (3.3)	26.8 (3.6)	26.8 (4.7)	25.2 (3.2)	25.8 (3.3)	24.8 (3.2)
BMI categories	Underweight (<18.5 kg/m^2^)	28 (0.8)	41 (0.5)	2 (0.2)	53 (1.9)	271 (0.4)	242 (0.2)	43 (0.75)
	Normal (18.5–<25 kg/m^2^)	1966 (54.8)	4308 (53.3)	345 (30.9)	953 (35.0)	33 502 (53.5)	42 952 (44.4)	3332 (58.4)
	Overweight (25–<30 kg/m^2^)	1372 (38.2)	3111 (38.5)	594 (53.3)	1137 (41.7)	24 529 (39.2)	43 888 (45.3)	1977 (34.6)
	Obese (≥30 kg/m^2^)	223 (6.2)	616 (7.6)	174 (15.6)	582 (21.4)	4335 (6.9)	9759 (10.1)	355 (6.2)
Smoking	Yes	…	3459 (37.9)	277 (24.9)	1021 (32.0)	26 242 (30.9)	27 803 (27.3)	…
Alcohol	None	…	449 (5.5)	…	…	…	2963 (4.1)	…
	Light drinking	…	4251 (51.8)	…	…	…	59 577 (82.3)	…
	Moderate/heavy drinking	…	3505 (42.7)	…	…	…	9882 (13.6)	…
Education	Low	190 (8.5)	2959 (25.9)	…	4299 (52.9)	17 069 (21.8)	4245 (4.4)	956 (16.6)
	Medium	398 (17.9)	6391 (55.9)	…	1115 (13.7)	28 230 (36.0)	43 576 (45.1)	2464 (42.8)
	High	1670 (73.9)	2079 (18.2)	…	2709 (33.3)	33 118 (42.2)	48 782 (50.5)	2335 (40.6)

Data are given as mean (SD) or number (percentage). Study numbers are based on singletons with data on at least one outcome and one exposure. Light smoking, <10 cigarettes per day; heavy smoking, ≥10 cigarettes per day; maternal light drinking, >0 and <3 units per week during pregnancy; maternal moderate/heavy drinking, ≥3 units per week during pregnancy; paternal light drinking, >0 and <7 units per week; paternal moderate/heavy drinking, ≥7 units per week. … Indicates data were not available; ABCD, The Amsterdam Born Children and Their Development Study; ALSPAC, Avon Longitudinal Study of Parents and Children; BASELINE, Cork Scope Baseline Study; BiB, Born in Bradford; BMI, body mass index; CHD, congenital heart disease; DNBC, Danish National Birth Cohort; MoBa, Norwegian Mother, Father and Child Cohort Study; and NINFEA, Nascita e Infanzia: gli Effetti dell'Ambiente (Birth and Childhood: Effects of the Environment).

* Chromosomal/genetic/teratogenic anomalies with a cause thought to be already known (see Table S3 for classifications).

^†^ Denotes that the study had data specifically during the first trimester. Numbers in the moderate/heavy columns for smoking and alcohol do not add up to the number of any smoking/alcohol because some studies used trimester‐specific data for the binary data, whereas moderate/heavy is an assessment of the exposure throughout pregnancy.

## Supporting information

Data S1–S4
Tables S1–S11
Figures S1–S32
References 51, 52
